# Editorial: Immunological biomarkers in response to Trypanosomatidae

**DOI:** 10.3389/fimmu.2024.1422067

**Published:** 2024-06-06

**Authors:** Soraya Gaze, Nayara I. Medeiros, Annette Dougall, Jacqueline Araujo Fiuza

**Affiliations:** ^1^ Group of Cellular and Molecular Immunology, Instituto Rene Rachou –Fiocruz, Belo Horizonte, MG, Brazil; ^2^ Department of Morphology, Federal University of Minas Gerais, Belo Horizonte, MG, Brazil; ^3^ Department of Agriculture, Fisheries and Forestry, Canberra, ACT, Australia

**Keywords:** leishmaniasis, Chagas Disease, genetic variation, immune response, neglected diseases

Chagas Disease and leishmaniasis are neglected parasitic diseases caused by protozoa of the family Trypanosomatidae. These two diseases mainly affect people in developing countries and millions of individuals are infected or live in endemic areas. Although *Leishmania* sp and *Trypanosoma cruzi* are intracellular parasites of the same family, their life cycles, intermediate hosts, and clinical outcomes are completely distinct. Moreover, different species of *Leishmania* cause different clinical outcomes, usually classified as cutaneous, mucocutaneous and visceral.

The clinical outcome of *Trypanosoma cruzi* infection is classified into asymptomatic, cardiac or gastrointestinal clinical forms. Despite the public health importance of leishmaniasis and Chagas Disease, there is still a lack of vaccines and/or new treatments for both.

In this Research Topic, contributing researchers have focused on different aspects of both diseases trying to understand the host immune response and how this response varies in different situations, including genetic variation.

In leishmaniasis, in its visceral clinical form caused by *Leishmania infantum*, CD4^+^ skin tissue-resident memory T cells have been shown to be important to the immune response. However, there is still a need for better characterization of this population to identify biomarkers to differentiate the prognosis outcome for the patient (Nateghi-Rostami and Sohrabi). Nevertheless, other players in the host immune system may influence the course of the disease. Genetic variation may explain part of the difference. A single nucleotide variation in IgG changes the amino acid from Histidine to Arginine and plays an important role in cytokine production, related to the amount and type, resulting in greater susceptibility to the development of visceral leishmaniasis (Cantarino et al.).

Chagas Disease also has many aspects that can influence the host response to the parasite. In this volume, a review shows that parasite genetic variation influences infectivity, reproduction, and vector variation. Therefore, a better understanding of the genetic variability of *Trypanosoma cruzi* could help to understand variations in the clinical forms of the disease and benefit the development of new treatments and/or vaccines.

Host genetic variation may also be important in parasitic diseases. Chagas Disease in its chronic form is classified according to cardiac inflammation and heart function failure (Silvestrini et al.). These inflammation markers are correlated to cardiac inflammation and differentially expressed genes in cardiac tissue are correlated with the presence of CD8+ T cells in the myocardium, an important T cell subset involved in cardiac tissue damage (Souza-Silva et al.).

Considering the development of a new diagnostic test, Enzyme-Linked Aptamer was shown, in an experimental mouse model, to be reliable for treatment comparison. This new diagnostic test appears to be a good way to screen new drugs before a pre-clinical trial development. Therefore, it can save time, effort, and funds. Hopefully, it can also be applied, in the future, to other neglected parasitic diseases (de Araujo et al.).

Trypanosomatid diseases, for all their complexities, also play different roles in experimental models compared to human disease. Thus, it is hard to transpose findings to clinical trials, not to mention that the same species of *Leishmania*, for example, can have different outcomes depending on the mouse strain. It is important to mention that the development of Artificial Intelligence can improve the search and development of biomarkers for Trypanosomatid disease, as well as others, by classifying all the important similarities and differences between the models studied. Therefore, there are many other aspects to explore in *Leishmania* and *Trypanosoma* infection, but this volume aims to take a small step toward understanding these diseases.

## Author contributions

SG: Conceptualization, Supervision, Writing – original draft, Writing – review & editing. NM: Conceptualization, Writing – review & editing. AD: Conceptualization, Writing – review & editing. JF: Conceptualization, Supervision, Writing – original draft, Writing – review & editing.

